# A Proof of Concept to Bridge the Gap between Mass Spectrometry Imaging, Protein Identification and Relative Quantitation: MSI~LC-MS/MS-LF

**DOI:** 10.3390/proteomes4040032

**Published:** 2016-10-26

**Authors:** Laëtitia Théron, Delphine Centeno, Cécile Coudy-Gandilhon, Estelle Pujos-Guillot, Thierry Astruc, Didier Rémond, Jean-Claude Barthelemy, Frédéric Roche, Léonard Feasson, Michel Hébraud, Daniel Béchet, Christophe Chambon

**Affiliations:** 1 Institut National de la Recherche Agronomique (INRA), Plateforme d’Exploration du Métabolisme (PFEM), F-63122 Saint Genès Champanelle, France; laetitiathrn@gmail.com (L.T.); delphine.centeno@inra.fr (D.C.); estelle.pujos-guillot@inra.fr (E.P.-G.); michel.hebraud@inra.fr (M.H.); 2 INRA, UMR 1019, Unité de Nutrition Humaine, CRNH Auvergne, F-63122 Saint Genès Champanelle, France; cecile.coudy-gandilhon@inra.fr (C.C.-G.); didier.remond@inra.fr (D.R.); daniel.bechet@inra.fr (D.B.); 3 Clermont Université, Université d’Auvergne, F-63000 Clermont-Ferrand, France; frederic.roche@univ-st-etienne.fr; 4 INRA Clermont-Ferrand Theix, UR370 Qualité des Produits Animaux, F-63122 Saint-Genès-Champanelle, France; thierry.astruc@inra.fr; 5 PRES Lyon, SNA-EPIS Research Unit, Exercise and Clinical Physiology Laboratory, University Hospital and Jean Monnet University, F-42023 Saint-Etienne, France; jc.barthelemy@univ-st-etienne.fr; 6 Unité de Myologie, LPE-EA4338, UJM/CHU, F-42055 Saint-Etienne, France; leonard.feasson@univ-st-etienne.fr

**Keywords:** MALDI mass spectrometry imaging, protein identification, label-free quantitation, skeletal muscle

## Abstract

Mass spectrometry imaging (MSI) is a powerful tool to visualize the spatial distribution of molecules on a tissue section. The main limitation of MALDI-MSI of proteins is the lack of direct identification. Therefore, this study focuses on a MSI~LC-MS/MS-LF workflow to link the results from MALDI-MSI with potential peak identification and label-free quantitation, using only one tissue section. At first, we studied the impact of matrix deposition and laser ablation on protein extraction from the tissue section. Then, we did a back-correlation of the *m*/*z* of the proteins detected by MALDI-MSI to those identified by label-free quantitation. This allowed us to compare the label-free quantitation of proteins obtained in LC-MS/MS with the peak intensities observed in MALDI-MSI. We managed to link identification to nine peaks observed by MALDI-MSI. The results showed that the MSI~LC-MS/MS-LF workflow (i) allowed us to study a representative muscle proteome compared to a classical bottom-up workflow; and (ii) was sparsely impacted by matrix deposition and laser ablation. This workflow, performed as a proof-of-concept, suggests that a single tissue section can be used to perform MALDI-MSI and protein extraction, identification, and relative quantitation.

## 1. Introduction

MALDI mass spectrometry imaging (MALDI-MSI) allows analyzing the spatial distribution of a wide variety of molecules simultaneously within a single tissue section [1]. MALDI-MSI is widely used to study biological phenomena thanks to its capability to map hundreds of molecules without any labeling, and in a single acquisition sequence. Previous investigations based on human biopsies indicated that muscle aging (sarcopenia) is associated with important modifications at the fiber-type level. Specifically, MALDI-MSI provided evidence for aging- and metabolic syndrome-dependent alterations in intra-myofibrillar lipids [2]. Using small human biopsies, MSI of proteins could be less sample-consuming than usual analytical techniques, such as two-dimensional gel electrophoreses [3] or shot-gun proteomics [4]. However, the main limitation of MALDI-MSI of proteins is the lack of direct identification, and therefore we need an additional step to overcome this issue [5]. Three strategies based on mass spectrometry methods allow protein identification: top-down and bottom-up experiments directly on tissue sections, and indirect identification approaches.

Bottom-up experiments on tissue sections consist of the tryptic digestion of proteins in spatially discrete regions followed by MALDI-MSI with MS/MS analysis [6]. However, on-tissue digestion of proteins may lead to partial diffusion of the resulting peptides and thus to miss-interpreting in situ protein localization [7,8]. Moreover, identifying proteins directly from tissues using MS/MS experiments for proteins or peptides is hampered by the low sensitivity caused by ion-suppression effects due to the complex molecular composition of tissues [5]. In addition, each protein analyzed by MALDI mass spectrometry results in a singly charged state ion, and ion activation by MALDI fails to produce fragmentation and identification. Protein sequence information could be achieved using MALDI In-Source Decay (ISD) mass spectrometry directly on tissue sections [9]. However, obtaining the protein sequence information by MALDI-ISD mass spectrometry is challenging in the case of protein mixtures [10]. Some studies focused on on-tissue tryptic digestion followed by micro-extraction [11] and on spatially directed micro-extraction of intact proteins [12] to identify proteins from specific locations in tissue. These strategies succeeded in identifying proteins from tissue sections, but their limitation lies in the need for at least a second tissue section for protein or peptide extraction.

Indirect identification approaches can be used to overcome these issues. These strategies are based on matching MALDI-MSI data with data generated using independent experiments, such as LC-MS/MS bottom-up approaches. Back-correlation of the *m*/*z* resulting from the off-tissue identified proteins to those of the proteins detected by MALDI-MSI is still a manual process, during which great care must be taken. Indeed, according to the mass tolerance window, thousands of possible proteins could match theoretically. Precautions must be taken, and additional validation might be necessary. Maier et al. [13] developed a method for the extraction and the identification of proteins embedded in the MALDI matrix layer, based on the principle that any MALDI-MSI biomarker must be contained in the MALDI matrix layer to be detectable by the mass spectrometer. This strategy has the great advantage to study the same set of proteins, both in MALDI-MSI and LC-MS/MS, after in situ extraction, and thus it reduces the risk of mistakes in protein identification. Thanks to this approach, these authors developed a publically available database, based on the identification of proteins extracted on matrix-coated tissue sections. Recently, Spraggins et al. [14] published a proof-of-concept study based on MALDI-FTICR-MSI of intact proteins linked with proteomic data obtained by LC-MS/MS. The use of high mass accuracy allowed them to identify proteins and protein modifications.

Nonetheless, to our knowledge, there is little information in the literature about the validation of the relative quantitation of proteins in MALDI-MSI. Different kinds of strategies at different tissue scales have been developed. At the biopsy scale, localized proteomic profiles and an overlay of the protein’s expression heat map on a tissue/biopsy were performed to give a relative quantitation of proteins within the tissue [15]. Another strategy consists of the quantitation-based mass spectrometry imaging of proteins by parafilm-assisted micro-dissection [16] at the tissue scale.

In our study, a workflow linking results from MALDI-MSI with potential protein identification and their relative label-free quantitation was developed. For this, we combined MALDI-MSI on a muscle section, protein extraction from the same section, their identification by LC-MS/MS, and then a back-correlation between label-free quantitation and peak intensities observed in MALDI-MSI. At first, we studied the impact of matrix deposition and laser ablation on protein extraction from tissue sections. Indeed, the MALDI procedure occurring during MSI acquisition might impact the extractability of proteins and thus the back-correlation between imaging and potential identification. Then, we did a back-correlation of the *m*/*z* of the off-tissue identified proteins by LC-MS/MS to those of the proteins detected by MALDI-MSI. This allowed us to compare the label-free quantitation of proteins based on peptide intensities with the proteins peak intensities observed in MALDI-MSI. This proof-of-concept was thus developed to study protein quantitation on a muscle section with the final objective of being used with regioselectivity. Finally, we validated and demonstrated the suitability of this workflow for the analysis of human muscle biopsies at the fiber-type level.

## 2. Materials and Methods

### 2.1. Experiment Summary

**Figure 1:**
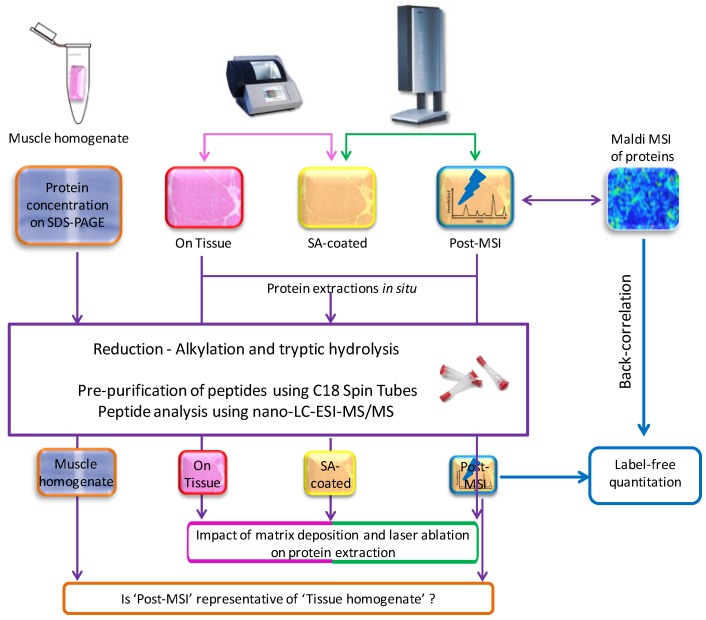
Summary of the experimental workflow.

The experimental workflow is described in Figure 1. For each of the eight individuals, serial muscle biopsy cross-sections were performed. Proteins were extracted directly on the section (‘On Tissue’), after matrix deposition (‘SA-coated’), and after spectra acquisition (‘Post-MSI’), and from the homogenization of 10 consecutive muscle sections (‘Muscle homogenate’). All extracts were hydrolyzed by trypsin, and the peptides were analyzed by LC-MS/MS. Proteins lists were compared to evaluate the impact of matrix deposition and laser ablation on protein extractability, and to evaluate how the extraction from the ‘Post-MSI’ is representative of the ‘Muscle homogenate’ reference approach. At last, back-correlation of *m*/*z* of ‘Post-MSI’ proteins to those of the proteins detected by MALDI-MSI, was performed to compare the label-free quantitation of ‘Post-MSI’ proteins with the peak intensities of MALDI-MSI proteins.

### 2.2. Reagents

All reagents were of the highest available grade. Water used in all experiments was purified by a Milli-Q system (Millipore, Milford, MA, USA). Acetonitrile and HPLC-grade methanol were purchased from Biosolve B. V. (Valkensvaard, Netherlands). Ammonium bicarbonate, ethanol, dl-dithiothreitol, iodoacetamide, isopentane, trifluoroacetic acid, urea, thiourea, CHAPS, protease inhibitor cocktail, and sinapinic acid (SA) were from Sigma (Saint-Quentin Fallavier, France). Sequencing grade modified porcine trypsin (V5111) was purchased from Promega (Charbonnières, France). Protein calibration standard was from Bruker Daltonics (Bremen, Germany). C18 Spin Tubes, and Agilent Peptide Cleanup were purchased from Agilent Technologies (Wilmington, DE, USA).

### 2.3. Subjects and Tissue Preparation

Our study included eight healthy young men (age 25 years) selected from Saint-Etienne University. They all underwent standard medical examination, and performed a maximal exercise stress before their inclusion in the study. The study was approved by the local Ethical Committee (ClinicalTrials.gov Identifier: NCT00759304), and all subjects provided written informed consent for participation. Needle biopsies were taken under local anesthesia from the right *vastus lateralis* muscle, after overnight fasting. Biopsies were mounted with tissue freezing medium, cryofixed in isopentane (−160 °C) cooled on liquid nitrogen and stored at −80 °C. Serial cross-sections (10 μm thick, 10 mm^2^) were performed using a cryostat (Microm, Francheville, France) at −20 °C.

### 2.4. MALDI Mass Spectrometry Imaging

For MALDI-MSI, sections were collected on conductive indium-tin-oxide glass slides (Bruker Daltonics, Bremen, Germany). Muscle sections were subjected to washing steps using 70% and 95% Ethanol to deplete lipids, and were dried in a desiccator for 30 min. The matrix was applied using the ImagePrep station (Bruker Daltonics) according to the protocol detailed in Table 1. The matrix was 10 mg/mL sinapinic acid in water/acetonitrile 60:40 (*v*/*v*) with 0.2% trifluoroacetic acid.

The MALDI spectra were acquired on an Autoflex Speed MALDI-TOF/TOF mass spectrometer with a Smartbeam laser using FlexControl 3.4 and FlexImaging 3.0 software packages (Bruker Daltonics). For protein imaging, ions were detected in positive linear mode at a mass range of *m*/*z* 2000–20,000 with a sampling rate of 0.63 GS/s. The lateral resolution was set to 100 μm and a total of 500 laser shots were accumulated per pixel at constant laser power. Deflection was set at *m*/*z* of 1500, and laser focus at medium. Analysis were performed using a detector gain of 2.783 V, ion source voltage 1 at 19.5 kV, ion source voltage 2 at 18.15 kV and lens voltage at 7 kV. A protein standard (Bruker Daltonics, Bremen, Germany) was employed for external calibration of spectra, which was done externally on the same target before each measurement.

### 2.5. ‘On Tissue’, ‘SA-Coated’, and ‘Post-MSI’ Sample Preparation

Proteins were extracted on serial muscle biopsy cross-sections: directly on tissue sections (‘On Tissue’), on matrix-coated muscle tissue (‘SA-coated’ for Sinapinic Acid), and on the sections used for MALDI-MSI measurements (‘Post-MSI’), using a method derived from Maier et al. [13]. Each experiment was repeated twice for each individual. For ‘On Tissue’, ‘SA-coated’, and ‘Post-MSI’ sections, the sample area was covered with 2 μL of 7.5% acetonitrile in 0.2% trifluoroacetic acid, and incubated for 1 min. The liquid containing the protein extract was collected, and this extraction was repeated once. The same area was then covered with 1 μL of 60% acetonitrile in 0.2% trifluoroacetic acid and the liquid containing the protein extract was immediately collected and combined with previous extracts (5 μL total). Protein extracts were reduced with 20 μL of 10 mM dithiotreitol in 50 mM ammonium bicarbonate (pH = 8), during 15 min at 55 °C. After cooling, alkylation was performed by adding 20 μL of 100 mM iodoacetamide in 50 mM ammonium bicarbonate, during 15 min at 20 °C in darkness. After neutralization, protein digestion was achieved by adding 25 μL of trypsin solution (20 ng/μL) in 50 mM ammonium bicarbonate, and overnight incubation at 37 °C.

Trypsin digestion was stopped with 2% trifluoroacetic acid, and after 5 min centrifugation at 3000× *g*, the supernatant was collected. The pellet was washed using 2% acetonitrile, 0.05% trifluoroacetic acid in water, sonicated for 5 min, centrifuged during 5 min at 3000× *g*, and the supernatant was collected and combined with the previous one, resulting in 100 μL samples.

### 2.6. ‘Muscle Homogenate’ Preparation

For ‘muscle homogenate’ experiments, 10 cross-sections were homogenized at 4 °C in a solubilization buffer containing 8.3 M urea, 2 M thiourea, 2% CHAPS, 1% dithiothreitol and protease inhibitor cocktail, and centrifuged for 10 min at 10,000× *g*. The supernatant was mixed with 1 volume 2% SDS, 5% β-mercaptoethanol, 10% glycerol and 62 mM Tris-HCl, pH 6.8, and heated at 95 °C for 5 min. SDS-PAGE (12% acrylamide) was performed using a Mini-Protean II electrophoresis unit (BioRad, Marnes-La-Coquette, France). Samples were loaded at 20 μg protein per lane. To concentrate the samples, gels were run at 100 V until the dye front reached the bottom of the concentration gel. Gels were fixed with 30% ethanol, 5% acetic acid, and stained overnight in Coomassie brilliant blue G-250. Excised lanes were reduced with 10 mM dithiotreitol, alkylated with 55 mM iodoacetamide (both in 50 mM ammonium bicarbonate), and incubated in 25 mM ammonium bicarbonate with acetonitrile (*v*/*v*) until destaining. After incubation in 100% acetonitrile, gel pieces were dried in a vacuum SpeedVac. They were further rehydrated with 30 μL of a trypsin solution (10 ng/μL in 25 mM ammonium bicarbonate), and finally incubated overnight at 37 °C. Peptide extraction was optimized by adding 24 μL of acetonitrile 100% followed by 10 min of sonication. The trypsin digests were dried in a vacuum SpeedVac and stored at −20 °C in a solution of 2% acetonitrile, 0.05% trifluoroacetic acid before LC-MS/MS analysis.

### 2.7. LC-MS/MS Sample Preparation, Data Acquisition, Database Search and Protein Identification

‘On Tissue’, ‘SA-coated’, ‘Post-MSI’ and ‘Muscle homogenate’ samples were desalted using C18 Spin Tubes (Agilent Peptide Cleanup, Agilent Technologies, Wilmington, DE, USA), according to the manufacturer’s instructions. Briefly, the resin was wet using 200 μL of 50% acetonitrile, the tubes were centrifuged at 1500 g for 1 min, and this step was repeated once. The resin was then equilibrated with 200 μL of 0.5% trifluoroacetic acid in 5% acetonitrile, tubes were centrifuged at 1500 g for 1 min, and this step was repeated once. Samples were then loaded on the resin, and the resin was washed three times using 200 μL of 0.5% trifluoroacetic acid in 5% acetonitrile. Samples were eluted using 20 μL of 70% acetonitrile with 0.1% trifluoroacetic acid, dried and resuspended in 0.05% trifluoroacetic acid for mass spectrometry analysis.

For Nano-LC-ESI-MS/MS analysis, peptides mixtures were analyzed in duplicate by online nanoflow liquid chromatography using the Ultimate 3000 RSLC (Dionex, Voisins le Bretonneux, France) with nanocapillary columns of 25 cm length × 75 μm I.D., 3 μm, 100Å (Acclaim PepMap100 C18, Dionex, Thermo Fisher Scientific, Waltham, MA, USA). The solvent gradient increased linearly from 4% to 50% acetonitrile in 0.5% formic acid at a flow rate of 200 nL/min for 100 min. The elute was electrosprayed in positive-ion mode at 2.7 kV in a LTQ-VELOS mass spectrometer (Thermo Fisher Scientific, Courtaboeuf, France) through a nanoelectrospray ion source which was operated in a CID top 10 mode (i.e., one full scan MS and the 10 major peaks in the full scan were selected for MS/MS). Full-enhanced-scan MS spectra were acquired with one microscan (*m*/*z* 400–1600). Dynamic exclusion was used with two repeat counts, 15 s repeat duration and 45 s exclusion duration. For MS/MS, isolation width for ion precursor was fixed at 2 *m*/*z*, single charged species were rejected; fragmentation used 37% normalized collision energy and a default activation of 0.250.

Thermo Proteome Discoverer v1.3 (Thermo Fisher Scientific, Waltham, MA, USA) was used for raw data file processing, and MASCOT was used for database search (http://www.matrixscience.com). For protein identification, the Uniprot Taxonomy Human (01/10/2013, 84,843 sequences) protein database was used. The following parameters were considered for the searches: peptide mass tolerance was set to 500 ppm, fragment mass tolerance was set to 0.5 Da and a maximum of two missed cleavages was allowed. Variable modifications were methionine oxidation (M) and carbamidomethylation (C) of cysteine. Protein identification was considered valid if at least one peptide with a statistically significant Mascot score assigned it (with Mascot score ≥ 36 for *p*-value < 0.05 with a False Discovery Rate (FDR) at 1%). Identification of proteins based on one peptide was accepted after checking the correct assignment of fragment ion matches (at least three consecutive fragments b/y, match peaks well above the background noise). Identifications not satisfying these defined criteria were rejected.

### 2.8. Label-Free Protein Quantitation

The spectra (Thermo raw files) acquired for ‘Post-MSI’ samples were loaded into the Progenesis QI software (version 1.0, Nonlinear Dynamics, Newcastle, UK) and label-free quantitation was performed as described by Theron et al. [4]. Briefly, for each sample, the profile data of the MS scans and MS/MS spectra were converted to peak lists comprising *m*/*z* and abundance with Progenesis QI using a proprietary algorithm. One sample was automatically set as the reference, and the retention times of all other samples were automatically aligned to create maximal overlay of the two-dimensional feature maps. Features with only one charge, with retention time windows lower than 6 s or with retention time lower than 20 min and higher than 80 min were excluded from further analyses. Using all remaining features, a normalization factor was calculated for each sample, to correct experimental variation. All unique validated peptides (with Mascot score ≥ 36 for *p*-value < 0.05) of an identified protein were included for quantification, and the total cumulative abundance was calculated by summing the abundances of all peptides allocated to the respective protein. Analysis was performed using the normalized abundances across all runs.

### 2.9. MALDI Data Analysis

Spectra from each imaging sequence were baseline-subtracted (TopHat) and smoothed (0.5 *m*/*z* Savitzky-Golay window, two cycles), and peaks were chosen (thresholds of 1.5 S/N, total possible peaks at 1000, peak width 1.0 Da) by a batch processing macro written in FlexAnalysis software (Version 4.3, Bruker Daltonics). Spectra normalization, using the Total Ion Count, and peak picking were performed using Scils Lab software (version 2016b, Scil GmbH, Bremen, Germany).

### 2.10. LC-MS Data Analysis

Compartments resource was used to determine and map the identified protein localization and get a confidence score [17]. The number of proteins within each subcellular localization was compared between each extraction procedure using a Khi square test.

### 2.11. Back-Correlation of MALDI and LC-MS Data

Back-correlation was performed to link the peaks observed by MALDI-MSI with the identification and label-free quantitation of proteins, using a 0.5% mass tolerance window. The correlation between quantitation of proteins by label-free LC-MS/MS (variable LF) and peak intensities of proteins observed by MALDI-MSI (variable MSI) was calculated using the formula: Correlation index (LF,MSI) = $\frac{\sum (LF-\bar{LF})\;\left(MSI-\bar{MSI}\right)}{\sqrt{\sum (LF-\bar{LF})²\sum \left(MSI-\bar{MSI}\right)²}}$.

## 3. Results

This study aimed at linking results from MALDI-MSI with potential peak identification and relative label-free quantitation obtained by LC-MS/MS. For this, we developed a new workflow combining MALDI-MSI on a muscle section, protein extraction ‘post-MSI’ from the same section, their identification by LC-MS/MS and their relative quantitation by label-free analysis. At first, we studied the impact of matrix deposition and laser ablation on protein extraction from tissue sections. Then, we did a back-correlation of the *m*/*z* of the ‘post-MSI’ proteins to those of the proteins detected by MALDI-MSI. This allowed us to correlate the label-free quantitation of proteins identified by LC-MS/MS with their peak intensities observed by MALDI-MSI.

### 3.1. Protein Extraction Procedures and Identification

**Figure 2:**
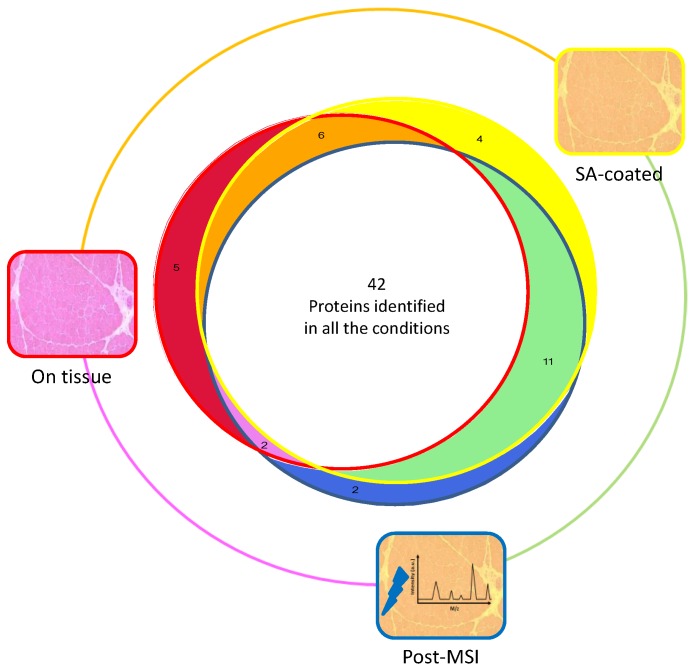
Venn diagram representing the number of proteins identified on muscle tissue section (‘On Tissue’, red circle), on sinapinic acid–coated section (‘SA-coated’, yellow circle), and after-MSI acquisition (‘Post-MSI’, blue circle); 42 proteins were common to all conditions, 4 were only identified in ‘SA-coated’ condition, 2 were only identified in ‘Post-MSI’ condition, 5 were only identified in ‘On-Tissue’ condition, 11 were common to ‘SA-coated’ (in green) and ‘Post-MSI’ conditions, 6 were common to ‘SA-coated’ and ‘On-Tissue’ conditions (in orange), 2 were common to ‘Post-MSI’ and ‘On-Tissue’ conditions.

The first milestone of the workflow of MSI~LC-MS/MS-LF is the protein extraction, since it is performed on matrix-coated sections after MALDI-MSI acquisition. To study the impact of matrix deposition and laser ablation on protein extraction and identification, we compared the identified proteins extracted on muscle tissue sections (‘On Tissue’) on sinapinic acid-coated sections (‘SA-coated’), and on sinapinic acid-coated sections after MALDI-MSI acquisition (‘Post-MSI’). The number of identified proteins in the 8 individuals was similar between the three conditions: 55, 63, and 55 after ‘On Tissue’, ‘SA-coated’, and ‘Post-MSI’ extractions, respectively, corresponding to a total of 72 proteins (Supplementary Data 1). In the present study, few proteins were specific to one condition, or were identified in only two conditions, but more importantly, 42 of them were shared by the three conditions (Figure 2). Therefore, regardless of the treatment, ‘On Tissue’, ‘SA-coated’ and ‘Post-MSI’ extractions resulted in a similar number of proteins. These results demonstrated that a bottom-up approach is possible after in situ extraction, and that the matrix deposition and laser ablation have no major impact on the number of identified proteins. Beyond the number of proteins identified in each condition, we wanted to make sure that the common 42 identified proteins corresponded to the most abundant peptides. The peptide spectrum match (PSM) can be used to evaluate the relative abundance of a protein within a sample. For each protein, we calculated an abundance index (%PSMs) as the percentage of the sum of PSMs for each condition (i.e., ‘On Tissue’, ‘SA-coated’, and ‘Post-MSI’) (Table 2 and Supplementary Data 1). The sum of abundances of the 42 proteins identified within the three conditions was 97% in ‘On Tissue’ extraction, 94% after ‘SA-coated’ extraction, and 94% after ‘Post-MSI’ extraction.

**Figure 3:**
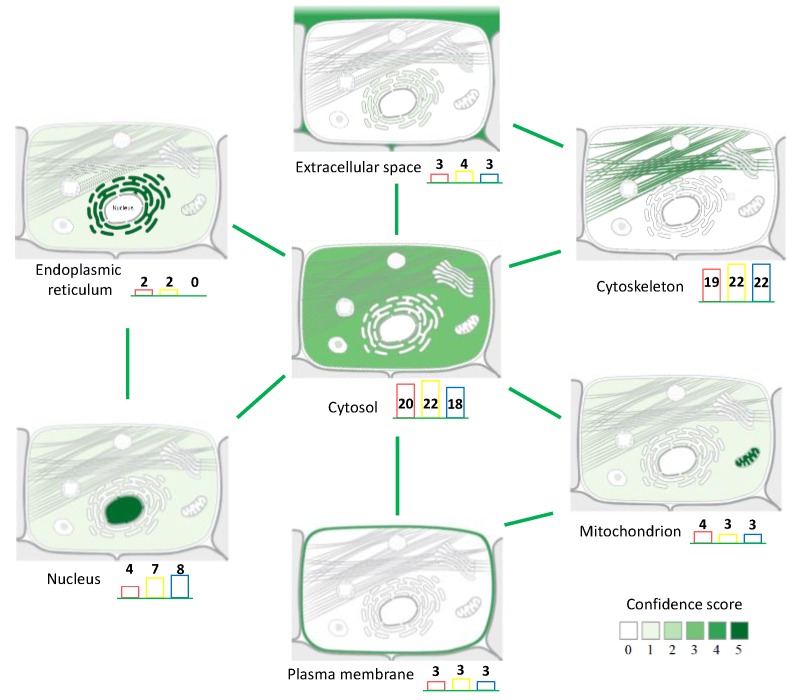
Main cellular localization of the identified proteins after ‘On Tissue’ (in red), ‘SA-coated’ (in yellow), and ‘Post-MSI’ (in blue) extraction procedures, mapped using Compartments [12]. Green lines indicate proteins belonging to several cellular compartments.

**Figure 4:**
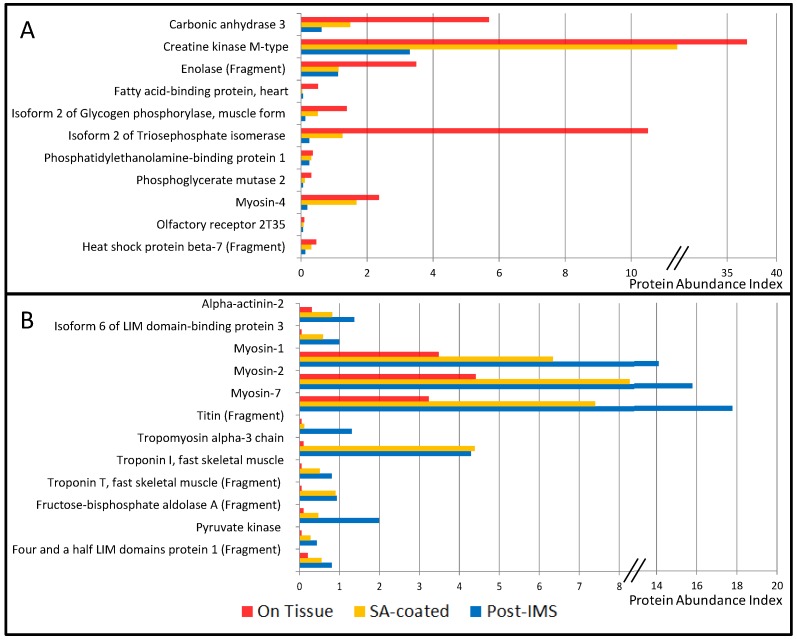
Representation of protein abundance index decreasing (**A**) and increasing (**B**) during the three extraction procedures (i.e., ‘On Tissue’ in red; ‘SA-coated’ in yellow; and ‘Post-MSI’ in blue).

Although matrix deposition and laser ablation did not impact the number of identified proteins, we wanted to verify that these procedures did not result in preferential extraction from a specific cellular compartment. To achieve this point, we analyzed the results using the Compartments resource [14] which maps the proteins within the cell and gives a confidence score to the localization evidence. The results showed that in situ extraction led to the identification of proteins from the cytosol, cytoskeleton, nucleus, mitochondrion, endoplasmic reticulum, plasma membrane, and extracellular space (Figure 3). All proteins were classified according to their main cellular compartment, but some of them are described in the database as belonging to several cellular compartments (Supplementary Data 2). However, although the cellular repartition was qualitatively similar for proteins from ‘On Tissue’, ‘SA-coated’ and ‘Post-MSI’ extracts, there were quantitative differences for some individual proteins. For the 42 proteins identified in the three extraction procedures, we compared the evolution of the abundance index to evaluate the proportion of proteins within each procedure. These revealed three groups: the first one corresponded to the proteins showing a decreasing abundance index during the extraction steps; the second one corresponded to the proteins showing an increasing abundance index during the extraction steps; and the third one corresponded to the proteins showing different kinds of patterns (Table 2, Figure 4). The first group included mainly cytosolic proteins: carbonic anhydrase 3 (CA3); creatine kinase M-type (CKM); enolase (fragment) (ENO3); fatty acid binding-protein, heart (FABP3); isoform 2 of glycogen phosphorylase, muscle form (PYGM); isoform 2 of triose phosphate isomerase (TPI1); phosphatidylethanolamine-binding protein 1 (PEBP1); phosphoglycerate mutase 2 (PGAM2). The second group included mainly structural proteins from the cytoskeleton and contractile apparatus: α-actinin 2 (ACTN2); isoform 6 of LIM domain-binding protein 3 (FHL1); myosin-1 (MYH1); myosin-2 (MYH2); myosin-7 (MYH7); titin (fragment) (TTN); tropomyosin α-3 chain (TPM1); troponin I fast skeletal muscle (TNNI2); troponin T fast skeletal muscle (fragment) (TNNT3). These results indicated that there was a lower abundance of cytosolic proteins and a higher abundance of structural proteins within the extracts after matrix deposition and laser ablation. Beside their subcellular location, other factors (such as their physicochemical properties) might also be important for the extractability of proteins from the matrix layer.

Protein identifications were compared to the databases MSiMass List [24] and MaTisse [13] (Table 2). MSiMass List is a public database implemented by users who assign identities to the peaks observed in their own experiment. MaTisse is a publically available database which compiles the identifications obtained in top-down and bottom-up approaches after in situ protein extraction. Most of our proteins were already described in a MALDI imaging experiment (Table 2). Among the 72 proteins identified in our experiment, only six were not described in these databases: Isoform 3 of kinesin-like protein KIF 15 (KIF15); Isoform 2 of aldo-keto reductase family 1 member B15 (ALDOC); Isoform 2 of DNA repair protein complementing XP-C cells (XPC); Isoform 3 of kinase d-interacting substrate of 220 kDa (KIDINS220); Olfactory receptor 2T35 (OR2T35); Phosphoglycerate kinase (PGK1). This result was independent of the extraction procedure as Isoform 2 of Aldo-keto reductase family 1 member B15 and Isoform 2 of DNA repair protein complementing XP-C cells were identified only on the ‘On Tissue’ section, Isoform 3 of Kinesin-like protein KIF15 only on the ‘SA-coated’ section, and Olfactory receptor 2T35 in the three procedures.

At last, it was important to determine how the ‘Post-MSI’ proteome is representative of the proteome studied by ‘Muscle homogenate’ proteomic workflow, i.e., using direct muscle homogenization and protein extraction. For this, we compared the identified proteins in both conditions (Supplementary Data 3). From the 195 proteins identified with the ‘Muscle homogenate’ workflow, 46 were found in the ‘Post-MSI’ proteome, and these 46 proteins accounted for 66% of the ‘Muscle homogenate’ PSMs. Regression analysis indicated that for these 46 proteins, there was a positive correlation (r = 0.83, *p* < 0.001) between their ‘Post-MSI’ and ‘Tissue Homogenate’ abundance indexes (%PSM).

### 3.2. Back-Correlation of the m/z of the Off-Tissue Identified Proteins to Those of the Proteins Detected by MALDI-MSI

The second milestone of the MSI~LC-MS/MS-LF workflow is the back-correlation of the proteins identified by LC-MS/MS to those detected by MALDI-MSI. Based on the idea from Maier et al. [13], that any MSI protein biomarker must be contained in the MALDI matrix layer to be detectable by the mass spectrometer, back-correlation relies on matching *m*/*z* identified by LC-MS/MS to *m*/*z* detected by MALDI-MSI.

**Figure 5:**
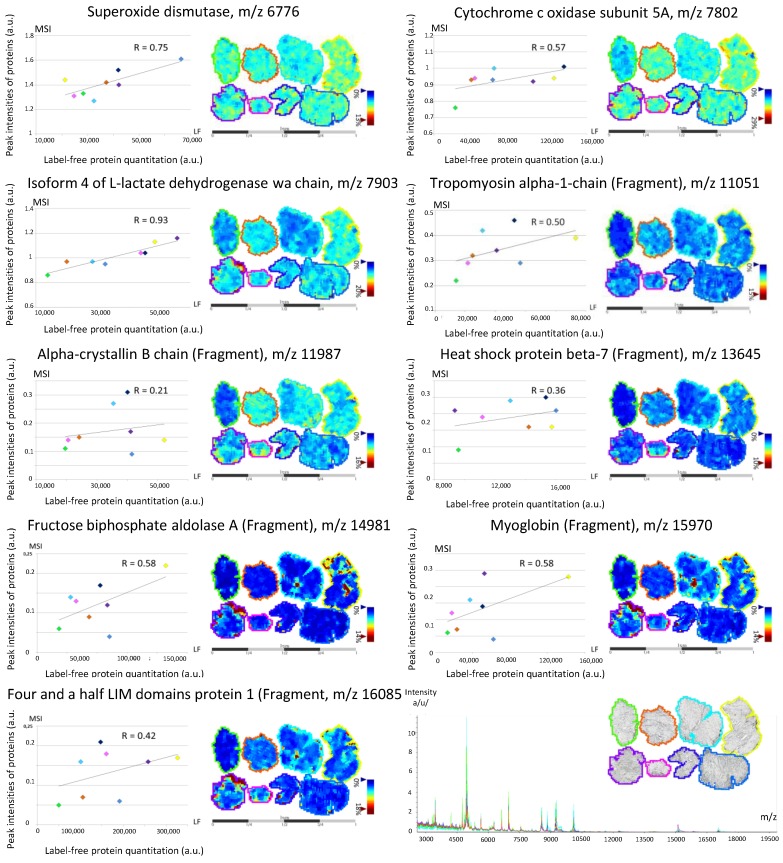
Mean spectra recorded from 2000 to 20,000 *m*/*z* on each muscle tissue section; a color is attributed to each of the eight individuals. Correlation between the mean intensity observed on the muscle section using mass spectrometry imaging (MSI) of the nine peaks back-correlated with protein identification and quantitation obtained by label-free (LF).

MALDI spectra were recorded from 2000 to 20,000 *m*/*z*, and a mean spectrum was calculated for each MALDI-MSI sequence. Mean spectra had a similar global pattern, and showed peaks with higher intensity at low mass range (Figure 5). We succeeded in linking protein identification to nine peaks observed by MALDI-MSI, using a mass tolerance interval set at 0.5% (Figure 5; Supplementary Data 4): superoxide dismutase (*m*/*z* 6776); cytochrome c oxidase subunit 5A, mitochondrial (*m*/*z* 7802); isoform 4 of l-lactate dehydrogenase A chain (*m*/*z* 7903); tropomyosin α-1 chain (fragment) (*m*/*z* 11051); α-crystallin B chain (fragment) (*m*/*z* 11,987); heat shock protein β-7 (fragment) (*m*/*z* 13,645); fructose-biphosphate aldolase A (fragment) (*m*/*z* 14,981); myoglobin (fragment) (*m*/*z* 15,970); and four and a half LIM domain protein 1 (fragment) (*m*/*z* 16,085).

The label-free strategy was then applied to compare quantitation of ‘Post-MSI’ proteins extracted in situ and their MALDI-MSI peak intensities. The analysis resulted in the quantitation of 35 proteins from the 57 identified in ‘Post-MSI’ (Supplementary Data 4), and gave a relative quantitation for all proteins linked to a peak observed by MALDI-MSI. We calculated the correlation between the label-free quantitation of ‘Post-MSI’ proteins and the peak intensity observed by MALDI-MSI (Figure 5). Except for α-crystallin B chain (fragment), which showed a correlation index of 0.21, we observed good correlations between both approaches: 0.36 for heat shock protein β-7 (fragment) and 0.42 for four-and-a-half LIM domain protein 1 (fragment); 0.50 for tropomyosin α-1 chain (fragment), 0.57 for cytochrome c oxidase subunit 5A, mitochondrial; 0.58 for both fructose-biphosphate aldolase C (fragment) and myoglobin (fragment); 0.75 for superoxide dismutase; and 0.93 for isoform 4 of l-lactate dehydrogenase A chain (with a significant result for the last two). These results demonstrated the relevance of the MSI~LC-MS/MS-LF workflow as a proof-of-concept, which aimed at linking MALDI-MSI peak intensities with label-free quantitation. To the best of our knowledge, this is the first time that such a study has been presented.

## 4. Discussion

At first, we studied the impact of matrix deposition and laser ablation on protein extraction from tissue sections. The number of proteins identified after extraction from tissue sections is consistent with what was found on sections of ocular lens where 50 to 100 proteins were identified [12], and after in situ hydrogel-based protein digestion on brain sections [18] where over 50 proteins were identified. The number of identified proteins is less than the mean number of proteins identified by Maier et al. [13], but they used tissue sections of about 5 cm², which is much larger than the ones used in the present work (10 mm^2^, see Experimental Section). Franck et al. [15] explained that the area of the tissue section is linked to the number of proteins identified and recommended to analyze sections of similar sizes to prevent any variations in protein number.

Mapping the main cellular compartment for each identified protein suggested an apparent homogeneity of the three extraction procedures: the statistical tests (Khi square test, Supplementary Data 2) indicated that none of the extraction procedures changed the qualitative distribution of the proteins between the different cellular compartments. Moreover, the distribution is consistent with that observed by Maier et al. [13] after in situ extraction and the bottom-up strategy. In addition, the repartition of proteins according to their cellular compartment is consistent with the characterization of the human *vastus lateralis* muscle by a global proteomic study [23]. This study succeeded in linking protein identification to nine peaks observed by MALDI-MSI. In terms of the ratio between detected ions and ions linked with identification, this is consistent with what was achieved on peptide back-correlation by Minerva et al. [25]. They identified 46 peptides of the 136 detected with MALDI-MSI, and for 31 of these, a back-correlation with LC-MS was possible and revealed a similar peak intensity ratio for 18 peptides.

Thanks to these results, we are able to determine the localization of these proteins, and to compare their intensities between different muscle sections to study various biological phenomena. Indeed, the proteins resulting from this analysis are known to be involved in several biological mechanisms occurring in skeletal muscle: aging [26,27,28,29,30,31,32], atrophy and myopathy [33,34,35] or association with non-obese type 2 diabetes [36].

## 5. Conclusions

The purpose of this proof-of-concept study was to evaluate the power of MALDI-MSI for relative quantitation of proteins between experimental conditions. The MSI~LC-MS/MS-LF workflow allowed us to use only one 10-μm-thick muscle section to perform both MALDI mass spectrometry imaging and protein extraction, with further back-correlation of label-free quantitation with the peak intensities observed in MALDI-MSI. This is critical for rare human biopsies. In a biological study, it should be recommended to perform analysis in triplicate to improve the accuracy of the label-free quantitation. Despite the use of low resolution for protein identification, we linked nine MALDI-MSI peaks to the label-free quantitation of proteins identified as biomarkers of various skeletal muscle physiological studies. We believe that this proof-of-concept could increase the potential application of MALDI-MSI of proteins in the study of skeletal muscle, and that it would be improved by the use of high resolution to increase the number of back-correlations between MALDI-MSI and protein identifications.

## Figures and Tables

**Table 1:** Individual ImagePrep phase settings for sinapinic acid (10 mg/mL in 60% ACN and 0.2% TFA) deposition onto tissue sections.

Phase	Sensor	Nebulization	Incubation	Drying
1	15 cycles	25% ± 30% power with fixed spray time of 2.2 s	15 s	50 s
2	0.1 V within 5–10 cycles	25% ± 30% power with 0.05 V sensor controlled spray time	30 s ± 30 s	Complete dry every cycle, safe dry 20 s
3	0.1 V within 6–18 cycles	25% ± 30% power with 0.10 V sensor controlled spray time		Grade 20% ± 60% complete dry every 2 cycles, safe dry 20 s
4	0.3 V within 12–40 cycles	25% ± 30% power with 0.2 V sensor controlled spray time		Grade 20% ± 60% complete dry every 4 cycles, safe dry 50 s
5	0.35 V ± 0.30 V, within 12–64 cycles	25% ± 35% power with 0.3 V sensor controlled spray time		Grade 20% ± 60% complete dry every 4 cycles, safe dry 60 s

**Table 2:** List of identified proteins extracted on muscle tissue section (‘On Tissue’), on sinapinic acid–coated section (‘SA-coated’), and on sinapinic acid-coated section after MALDI MSI acquisition (‘Post-MSI’), expressed as the percentage of the sum of PSMs for each procedure condition. References correspond to previous studies identifying proteins in a MALDI-MSI experiment. The main cellular compartments are: C, cytosol; Ck, cytoskeleton; ER, endoplasmic reticulum; ES, extracellular space; M, mitochondria; Mb, plasma membrane; N, nucleus.

Symbol	Accession	Description	Abundance Index (%)			References	Main Cellular Compartment
			On Tissue	SA-Coated	After-MSI		
ACTA1	P68133	Actin, α skeletal muscle	4.31	3.01	3.42	[9]	Ck
ACTN2	P35609	A-actinin-2	0.31	0.82	1.37	[9]	Ck
ATP5B	P06576	ATP synthase subunit β, mitochondrial (Fragment)	0.15	0.16	0.12	[9]	Mb
CA3	P07451	Carbonic anhydrase 3	5.69	1.49	0.62	[9]	C
CKM	P06732	Creatine kinase M-type	37.0	11.4	3.29	[9]	C
DES	P17661	Desmin	0.10	0.08	0.44	[9]	Ck
ENO3	P06733	Enolase (Fragment)	3.49	1.14	1.12	[9]	C
FABP3	P05413	Fatty acid-binding protein, heart	0.51	0.04	0.06	[9]	C
FHL1	Q5JXI0	Four and a half LIM domains protein 1 (Fragment)	0.21	0.55	0.81	[9]	N
ALDOA	P04075-H3BR68	Fructose-bisphosphate aldolase A (Fragment)	0.10	0.47	1.99	[9]	C
ALDOC	P09972	Fructose-bisphosphate aldolase C (Fragment)	0.05	0.39	0.06	[9]	C
GAPDH	P04406	Glyceraldehyde-3-phosphate dehydrogenase	0.97	1.45	0.12	[9]	C
HSPB1	P04792	Heat shock protein β-1	0.10	0.59	0.12	[9]	N
HSSPB7	C9J5A3-E9PN25	Heat shock protein β-7 (Fragment)	0.46	0.31	0.12	[9]	N
HBA1	P69905	Hemoglobin subunit α	0.26	2.11	0.56	[16]	ES
HBB	P68871	Hemoglobin subunit β	1.13	4.46	2.05	[16]	ES
PYGM	P11217-2	Isoform 2 of Glycogen phosphorylase, muscle form	1.38	0.51	0.12	[9]	C
TRDX	P10599-2	Isoform 2 of Thioredoxin	0.26	0.08	0.19	[17]	C
TPI1	P60174-1	Isoform 2 of Triosephosphate isomerase	10.51	1.25	0.25	[9,16]	C
LDHA	P00338-4	Isoform 4 of L-lactate dehydrogenase A chain	0.05	0.16	0.06	[9]	C
LDB3	O75112-6	Isoform 6 of LIM domain-binding protein 3	0.05	0.59	0.99	[9]	Ck
MB	B0QYF8	Myoglobin (Fragment)	4.72	4.66	3.67	[9]	C
MYBPC1	G3V1V7	Myosin binding protein C, slow type, isoform CRA_e	0.05	0.23	0.19	[9]	C
MYL1	P05976	Myosin light chain 1/3, skeletal muscle isoform	5.84	6.73	1.86	[9]	Ck
MYL3	P08590	Myosin light chain 3	1.18	2.47	1.86	[9]	Ck
MYLPF	Q96A32	Myosin regulatory light chain 2, skeletal muscle isoform	0.56	1.57	0.68	[9]	Ck
MYH1	P12882	Myosin-1	3.49	6.34	14.11	[9]	Ck
MYH2	Q9UKX2	Myosin-2	4.41	8.26	15.79	[9]	Ck
MYH4	Q9Y623	Myosin-4	2.36	1.68	0.19	[9]	Ck
MYHCB	P12883	Myosin-7	3.23	7.40	17.78	[9]	Ck
NEB	F8WCL5	Nebulin	0.05	0.04	0.19	[9]	Ck
OR2T35	Q8NGX2	Olfactory receptor 2T35	0.10	0.08	0.06		Mb
PEBP1	P30086	Phosphatidylethanolamine-binding protein 1	0.36	0.31	0.25	[9,15]	C
PGAM2	P15259	Phosphoglycerate mutase 2	0.31	0.12	0.06	[9]	C
PKM	H3BQ34	Pyruvate kinase	0.05	0.27	0.44	[9]	C
TTN	Q8WZ42	Titin (Fragment)	0.05	0.12	1.31	[9]	Ck
TPM1	P09493-H0YK20	Tropomyosin α-1 chain (Fragment)	0.77	6.03	5.22	[9]	Ck
TPM3	P06753	Tropomyosin α-3 chain	0.10	4.39	4.29	[9]	Ck
TPM2	P07951	Tropomyosin β chain	1.74	10.02	5.90	[9]	Ck
TNNC2	P02585	Troponin C, skeletal muscle	0.36	0.94	0.44	[9]	Ck
TNNI2	P48788	Troponin I, fast skeletal muscle	0.05	0.51	0.81	[9]	Ck
TNNT3	C9JCA5	Troponin T, fast skeletal muscle (Fragment)	0.05	0.90	0.93	[9]	Ck
IFIT2	P09913	Interferon-induced protein with tetratricopeptide repeats 2	0.15	0.04		[9]	ER
IDH2	B4DFL2	Isocitrate dehydrogenase [NADP] (fragment)	0.10	0.16		[9]	M
KIDINS220	Q9ULH0-3	Isoform 3 of Kinase D-interacting substrate of 220 kDa	0.05	0.04			Mb
PGM1	P36871	Phosphoglucomutase-1	1.49	0.08		[9]	C
S100A1	P23297	Protein S100-A1	0.15	0.20		[9,16]	ER
ALB	P02768	Serum albumin	0.15	0.16		[9,15]	ES
COX5A	H3BRM5	Cytochrome c oxidase subunit 5A, mitochondrial	0.05		0.06	[9]	M
SOD2	B4E3K9-H7BYH4	Superoxide dismutase	0.15		0.12	[9]	M
GSTP1	A8MX94	Glutathione S-transferase P	0.05			[9,15]	C
AKR1B15	C9JRZ8-2	Isoform 2 of Aldo-keto reductase family 1 member B15	0.10				C
XPC	Q01831-2	Isoform 2 of DNA repair protein complementing XP-C cells	0.05				N
HEBP2	Q9Y5Z4-2	Isoform 2 of Heme-binding protein 2	0.10			[9]	M
PGK1	B7Z7A9	Phosphoglycerate kinase	0.46				C
PFKM	P08237	6-phosphofructokinase, muscle type (Fragment)		0.04	0.06	[9]	C
HSPB5	E9PR44-E9PNH7	A-crystallin B chain (Fragment)		0.23	0.37	[9]	N
COL1A2	P08123	Collagen α-2(I) chain		0.04	0.06	[9]	ES
HIST1H1T	P22492	Histone H1t		0.12	0.12	[15,18]	N
H2AFV	A8MQC5	Histone H2A		0.04	0.06	[9]	N
FNC	Q14315-2	Isoform 2 of Filamin-C		0.04	0.25	[9]	C
MYOZ1	Q9NP98	Myozenin-1		0.31	0.25	[9]	N
SERCO1	B3KY17	Sarcoplasmic/endoplasmic reticulum calcium ATPase 1		0.04	0.25	[9]	Mb
TPM3	Q5VU72	Tropomyosin 3, isoform CRA_a		3.37	2.86	[9]	Ck
TNNC1	P63316	Troponin C, slow skeletal and cardiac muscles		0.31	0.68	[9]	Ck
TNNI1	P19237	Troponin I, slow skeletal muscle		0.27	0.75	[9]	Ck
CASQ1	P31415	Calsequestrin-1		0.08		[9]	M
CSRP3	P50461	Cysteine and glycine-rich protein 3		0.12		[9]	C
KIF15	Q9NS87-3	Isoform 3 of Kinesin-like protein KIF15		0.12			C
USMG5	Q96IX5	Up-regulated during skeletal muscle growth protein 5		0.08		[9]	M
UQCRB	P14927	Cytochrome b-c1 complex subunit 7			0.06	[1]	M
UBB	J3QSA3	Ubiquitin (Fragment)			0.12	[9,15,16]	N

## Supplementary Materials

The supplementary materials are available online at www.mdpi.com/2227-7382/4/4/32/s1.

## Abbreviations

MALDI: Matrix Assisted Laser Desorption Ionization
LC-MS: Liquid Chromatography – Mass Spectrometry
LF: Label-Free
MSI: Mass Spectrometry Imaging
ISD: In-Source Decay
SA: Sinapinic Acid
ACN: Acetonitrile
TFA: Trifluoroacetic Acid
PSM: Peptide Spectrum Match
C: Cytosol
Ck: Cytoskeleton
ER: Endoplasmic Reticulum
ES: Extracellular Space
M: Mitochondria
Mb: Plasma Membrane
N: Nucleus
